# Impact of periodic intensification of routine immunization within an armed conflict setting and COVID-19 outbreak in Cameroon in 2020

**DOI:** 10.1186/s13031-022-00461-1

**Published:** 2022-06-02

**Authors:** Andreas Ateke Njoh, Yauba Saidu, Hassan Ben Bachir, Shalom Tchokfe Ndoula, Eric Mboke, Raoul Nembot, Afizu Chrakoh Tambasho, Messang Blandine Abizou, Judith Seungue, Clarence Mbanga, Victor Njie Mbome

**Affiliations:** 1 Expanded Program On Immunization, Central Technical Group EPI, Ministry of Public Health, Messa, P.O. Box 2084, Yaoundé, Cameroon; 2 School of Global Health and Bioethics, Euclid University, Bangui, Central African Republic; 3 Clinton Health Access Initiative, Yaoundé, Cameroon; 4 Institute for Global Health, Siena University, Siena, Italy; 5 Ministry of Public Health, Yaounde, Cameroon; 6 Faculty of Medicine and Biomedical Sciences, University of Yaounde 1, Yaoundé, Cameroon; 7 Faculty of Health Science, University of Buea, Buea, Cameroon

**Keywords:** Periodic intensification of routine immunization, Armed conflict, COVID-19, South west region, Cameroon

## Abstract

**Introduction:**

Cameroon’s Southwest Region (SW) has been hit by an armed conflict for over half a decade now, negatively affecting the region’s routine immunization and disease surveillance activities. This negative effect was further acerbated by the COVID-19 pandemic, which alongside the conflict, caused thousands of children to miss out on life-saving vaccinations. Herein, we present the contribution of periodic intensification of routine immunization in improving immunization and surveillance activities amid crises.

**Method:**

Periodic intensification of routine immunization (PIRI) and disease surveillance were carried out in three rounds per health district. Before the intervention, the security profile of each district involved was reviewed. Data for this study was extracted on vaccination and surveillance activities from the District Health Information Software and monthly regional reports for 2019 and 2020 from the SW delegation of health.

**Results:**

54,242 persons were vaccinated in the SW following these interventions. An increase in performance was observed in all 18 health districts in 2020 compared to 2019. Both DPT-HebB-Heb-3 vaccine and OPV-3 coverage rose by 28% points. Similarly, the proportion of health districts that investigated at least a case of acute flaccid paralysis increased by 83%, rising from just three districts in 2019 to all 18 in 2020.

**Conclusion:**

PIRI was a practical approach to improving vaccination coverage and surveillance indicators in this region amidst the ongoing armed conflict and COVID-19 pandemic.

## Introduction

Immunization is a global health and development success story contributing to human health and wellbeing worldwide [1–3]. This discipline saves millions of lives each year. It helps populations across the globe to live longer and enjoy healthier lives [3]. There is no better depiction of the immunization success story than global efforts in Africa, which resulted in the African Region being declared free of the wild poliovirus in August of 2020 [4]. Indeed, this African Region has registered remarkable progress in disease control ever since the Expanded Program of Immunization (EPI) was set up in the mid-1970s. EPIs have successfully raised vaccine coverage for many pediatric vaccines from below 3% in the mid-70 s to nearly 80% in 2019 [5, 6].

Despite this progress, coverage for many of these antigens has plateaued and slipped back in many countries in the region. This stagnation or regression in coverage leaves almost 10 million children vulnerable to vaccine-preventable diseases (VPDS) [6]. Armed conflicts and the COVID-19 pandemic further heightened this vulnerability. COVID-19 has caused unparallel disruptions in health systems across several countries, impacting both the demand and supply sides of immunizations [6, 7]. In fact, in 2020, COVID-19 led to the cancellation of over a dozen vaccination campaigns in the region [6]. The result was almost 8 million African children missed out on the first dose of Diphtheria, Pertussis, Tetanus Hepatitis B, and Hemophilus influenza B vaccine (DPT-HepB-Hib-1 or Penta-1) in 2020—almost twice the figure recorded in 2019, and further 2 million children missing out on the Penta-3 [9].

Cameroon recorded its first case of COVID-19 in March 2020 in the capital city, Yaoundé [10]. The disease’s rapid spread to other regions led to significant travel restrictions in the same month to limit its propagation within the country [11]. These restrictions to gatherings included the prohibition of major health interventions like mass vaccination activities. The rules led to suspending the March 2020 national immunization against poliomyelitis. The COVID-19 outbreak caused a dramatic decline in the utilization of health services, including routine vaccination services in many regions of the country [12–14]. In the SW, COVID-19 worsened the negative impact of the prevailing armed conflict.

Armed conflicts commonly interfered with the normal functioning of affected communities. Conflicts lead to population movement, looting of infrastructures, and population displacement. In 16 countries affected by armed conflict across the globe, armed violence favored vaccine scarcity in affected communities, partly due to challenges in shipment and closure of health facilities following the insecurity [15]. Therefore, this insecurity reduces the uptake of life-saving vaccines in most settings and exposes the population to outbreaks [15–17]. After independence, Cameroon experienced peace for several decades. In 2014, the Boko Haram crisis erupted in the Far North Region, leading to distortion of the functioning of this community [18]. By late 2016 sociopolitical crisis erupted in the North West Region and SW of the country. The crisis in these two English-speaking regions later degenerated into a full-blown up armed conflict. The conflict resulted in the killing of health workers, looting of health facilities, distortion of health service delivery, and a drop in all EPI indicators [19]. These have harmed health service delivery in the region, with immunization services being particularly affected. In fact, in a recently published paper, we reported that the region had experienced a 42% drop in DPT-3 coverage between 2016 and 2019 [19]. This crisis was also associated with the deterioration of the surveillance indicators of the significant vaccine-preventable diseases (measles, yellow fever, polio, and neonatal tetanus) [19]. A surge in vaccine-preventable conditions and low vaccine uptakes are associated with armed violence and vaccine hesitance to life-saving vaccines in this setting [20, 21].

The EPI carries out routine vaccination and surveillance of major childhood diseases. The series of vaccines administered by the program primarily covers the period from birth to 9 months [18, 21]. The vaccines that were commonly administered to the population by the EPI in this country are reported in the study by Saidu 2021 [19]. However, the package of vaccines used by the EPI program may include more vaccine types and even an extended age group depending on the global and country needs. Periodic intensification of routine immunization (PIRI) is an intermittent intervention within a time limit [22, 23]. These interventions reinforce routine immunization of unvaccinated or under-vaccinated populations. During PIRI, participants’ eligibility is screened based on their ages and vaccination history [22, 23]. The vaccination team then recorded the dose of vaccines administered [22]. PIRI usually takes the form of child health weeks, days, or months. During this period, the population is sensitized on the importance of vaccination and its role in disease prevention [22]. These interventions help the health workers get close to the target population, and it is helpful to reinforce disease surveillance and actively search for cases of vaccine-preventable diseases.

With the worsening trend in vaccination coverage and other indicators of the EPI in this region, there was a need to carry out interventions to improve these health indicators. The ministry of health and its partners decided to implement PIRI to limit outbreaks of VPDs in the region [14]. An assessment of PIRI’s contribution to overall vaccination coverage and surveillance indicators in the SW has not been reported. As such, we set out to assess the impact of this PIRI on vaccination coverage and disease surveillance in the region.

## Materials and method

### Study design

This was a cross-sectional study carried out from March through August 2021, using data on vaccination and surveillance activities from all 18 health districts of the SW. This study design enabled the researchers to explore the impact of PIRI on immunization coverage and disease surveillance activities in the SW amid the ongoing sociopolitical crisis and the COVID-19 pandemic.

### Study area

This study was conducted in the SW of Cameroon. The region is home to over two million persons with diverse cultural and traditional backgrounds. Over 44% percent of its inhabitants reside in rural areas and are mainly involved in activities like farming and fishing. This region was hit by insecurity in late 2016, resulting in the mass displacement of a considerable proportion of its population. The conflict has also resulted in the looting and destruction of health facilities, abduction and killing of health workers, and distortion of the health system in the region [19, 20]. On April 2, 2020, the region recorded its first case of COVID-19. The COVID-19 cases have been increasing with a peak between epidemiologic week 20 and 25 of 2020 [26].

The region consists of 18 health districts led by a district medical officer (DMO). Each health district is further sub-divided into health areas (116 health areas in total for the region), led by a chief of health area. The head of the health area is by default the manager of the leading health facility within the health area. Each of these health areas has at least one health facility, public or private [19].

### Organization of immunization services

**Fig. 1:**
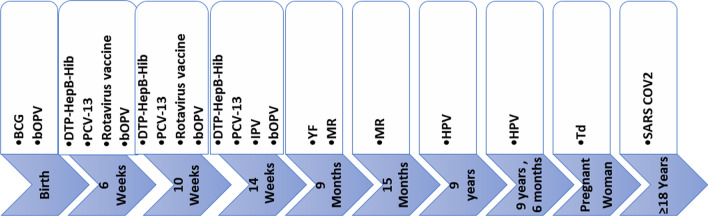
EPI Immunization schedule in Cameroon. This figure illustrates the vaccination calendar of Cameroon. The timeframe under each vaccine indicates the age from birth at which the vaccine is authorized to be administered to the target. For pregnant women who received the tetanus vaccine in childhood, three doses of Td are administered. The first dose at contact and the next is given one month after, and the third dose is given six months after the third dose. For women who did not receive a tetanus vaccine in childhood, a total of five doses are administered. After the first three doses above, a fourth dose is given 1 year later and the fifth dose 1 year after the fourth. BCG: Bacilli, Calmette Guerin, bOPV: bivalent Oral Polio Vaccine, DPT-HepB-Hib: Diphtheria, Pertussis, Tetanus Hepatitis B and Hemophilus influenza B, HPV: Human Papilloma Virus, IPV: Inactivated Polio Vaccine, MR: Measles and Rubella, PCV: Pneumococcal Conjugate Vaccine, SARS COV 2: Severe Acute Respiratory Syndrome Corona Virus two, Td: Tetanus Dyphteriam, YF: Yellow Fever

A lead health facility in each health area stores vaccines and coordinates vaccination and other EPI activities in the zone. This facility also ensures reporting in the area using the District Health Information Software (DHIS2) and other paper-based weekly and monthly tools. Vaccines are administered through the EPI in Cameroon as initially described [19]. In March 2020, the second dose of the Measles-Rubella (MR2) vaccine was introduced and administered at 15 months. Also, from October 2020, the vaccine against the Human Papilloma Virus (HPV) was introduced to be issued to adolescent girls 9 years old [27], and the second dose is administered six months after the first (Fig. 1). From April 2021, to curb the spread of COVID-19, the vaccine against severe acute respiratory syndrome coronavirus 2 (SARS COV2) was introduced for persons aged 18 years and above [28] (Fig. 1). According to national guidelines, these vaccines are administered to all eligible persons at the vaccinating health facility daily and in outreach sites, where feasible, at no cost.

Mindful of the harm caused by COVID-19 and the insecurity experienced in this region, the EPI manager of the SW (under the supervision of the EPI Central Technical Group) engaged in the intensification of immunization and surveillance activities in the health districts of the SW through the sponsorship of the United Nations Children Funds (UNICEF). PIRI was carried out in three rounds in each health district. Each round was separated from the next by 28 to 30 days. The interventions took place in March, May, June, November, and December 2020, and the last phase was in January 2021. April 2020 was skipped following the measures put in place to control the COVID-19 outbreak in the country. This period also was used to elaborate guidelines to ensure continuity of vaccination services amid COVID-19. The onset of PIRI involved three days of sensitization. These three days were followed immediately by three other days of vaccination and active case search in the community for VPDs such as poliomyelitis, measles, yellow fever, and neonatal tetanus. Health workers paid more attention to poliomyelitis surveillance through AFP case searches. The search preference was stimulated by the fact that the country strived to be certified free of wild poliovirus in 2020.

### Interventions before the PIRI

Before 2020, there was no clear strategy to reach infants in this insecure region. So, when possible, EPI activities were carried out as routine activities within the existing health facilities. With the worsening indicators, a commission was set up by the ministry of health in late 2019 to study and elaborate strategies to improve EPI indicators in this region. Before the onset of PIRI, the regional EPI program manager and the district medical officers (DMOs) did an assessment. They set up strategies for a successful PIRI amid insecurity and COVID-19. Firstly, each health district came up with a micro plan. They estimated the number of vaccines and consumables needed, mapped sites where internally displaced persons (IDPs) could be found, and estimated the number of human resources required and intervention cost.

Then, the district and regional teams assessed the level of insecurity within each health area of the respective health districts. The team equally anticipated the most probable days when violence could erupt or curfews could be announced by the government or the non-state armed forces groups. Periods avoided included national public holidays, major state events like elections, and trial days for arrested leaders of the non-state arm groups. These guided planning the most likely time to organize PIRI in each district or health area. Commodities were then pushed from the region to the districts and health facilities using the safest means of transport during a safe period per axis of shipment. In addition, all health workers (community health workers and health facility staff) used to carry out PIRI in a given community were physically healthy and permanently residing in those communities. These health workers were briefed on their respective tasks and deployed to work within the communities. Before the day of the onset of the activity, community actors were used to advocating with local opinion leaders. Secondly, announcements on the period of PIRI and the commodities to be administered were made in gatherings like churches, mosques, and markets. Town criers also made announcements, especially in the rural communities, and the radio was equally used where available. Three days before the start of PIRI, communication activities were intensified in every community daily.

### Interventions during PIRI

Following the national guideline on continuity of immunization in COVID-19, each health worker used a face mask and had a hand disinfecting solution. The central strategy used was outreach in communities and in sites where IDPs were identified to be based. Vaccination activities were equally continued in health facilities. Each vaccination team was made up of a vaccinator, a recorder, and a mobilizer. Door-to-door was the primary strategy within the community, and the vaccination team that transported the vaccine through a vaccine carrier ensured that all vaccination activities were carried out in the shade outside of the homes visited. Commodities administered included vaccines, vitamin A and Mebendazole to infants, adolescent girls, and pregnant women. Before issuing any commodity, the individual’s age, vaccine receipt, and pregnancy status were verified. The missed vaccine doses were administered. Vitamin A and Mebendazole were systematically administered to every child under 5 years. After distributing the commodities, the recorder tallied the information on a sheet. During the PIRI, the vaccination teams actively searched for missed cases of acute flaccid paralysis (AFP), and suspected cases of measles, yellow fever, and neonatal tetanus. If any case was identified, the investigation form was filled out. The infant was guided to the health facility, where the specimen was collected as indicated and forwarded to Yaounde for analysis at Centre Pasteur.

### Interventions after PIRI

At the end of each day, an evaluation was done at the respective health areas. The number of persons who benefitted from the interventions was synthesized and sent to the district health service. During this health area evaluation, corrective measures were made to improve performance and ensure the safety of the workers for the subsequent days. At the end of each round of PIRI, the respective health districts had an evaluation meeting with the health area leads (chief of health areas and focal communication persons). At the end of the district evaluation, a synthesis of the results obtained per health area was made and shared with the regional EPI team. Each health facility in the health area entered the data alongside other health interventions at the end of the month into the Dhis2.

Once PIRI was adopted as the strategy of choice, funds were mobilized by the ministry of health through UNICEF. The cost per child was calculated by dividing the total amount of money spent for all the rounds of PIRI in 2020 by the number of persons vaccinated.

### Data source and analysis

The data for this study were retrieved from the standard EPI data reporting tool, the Dhis2, for 2019 and 2020. According to common practice, this platform is updated monthly with data from health facilities in each district for the corresponding period.

Also, monthly reports from the SW delegation of health were used to obtain surveillance data. In contrast, a pretested questionnaire was used to abstract key variables, including the security profile of the health districts and the approach and time of implementation of PIRI. These data were extracted for 2019 till 2020, during which PIRI was carried out amidst insecurity and the COVID-19 outbreak in the region. The EPI lead of the region filled out the questionnaires. The investigation team then resolved any identified data discrepancy by directly calling the regional head or the district medical officers concerned.

Data obtained were analyzed using Microsoft Office Excel 2019. Summary statistics were used to present findings. Bar charts and line graphs were used to display trends and compare EPI vaccination performance across the years investigated.

## Results

At the end of the intensification of routine immunization and surveillance activities in the region, 54,242 persons benefitted from at least one catchup dose of a missed vaccine during the intervention. Contrary to 2019, when just 24,751 infants received Penta-3, in 2020, 37,407 children were successfully vaccinated with this vaccine. This activity also improved the number of persons who received vaccines that were newly introduced in the country’s EPI in 2020 (MR2 at 15 months and HPV vaccine at 9 years) (Fig. 1). The cost per person vaccinated in 2020 during the PIRI was 4.50 USD.

**Fig. 2:**
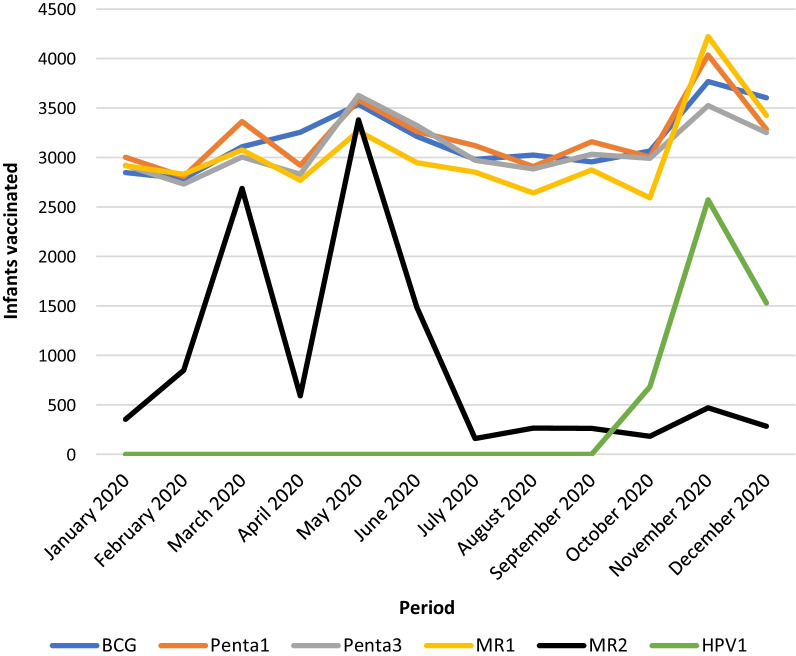
Trends in the number of infants vaccinated and reported in Dhis 2 in 2020. The line graph presents the trend in the total number of infants or adolescents vaccinated with the third dose of DPT (Penta), first and second dose of Measles-Rubella vaccine, and the first dose of Human Papilloma Virus Vaccine (for adolescent girls). The blue line represents the infants vaccinated with DPT-3 (Penta 3). The brown represents those immunized with the first dose of the Measles-Rubella vaccine. Meanwhile, the black line represents the infants vaccinated with the second dose of the Measles-Rubella vaccine. Finally, the yellow line represents the infants vaccinated with the first dose of the Human Papilloma Virus vaccine (introduced 12 October 2020). The spikes represent the periods of PIRI, while the lull represents the period of routine immunization

**Fig. 3:**
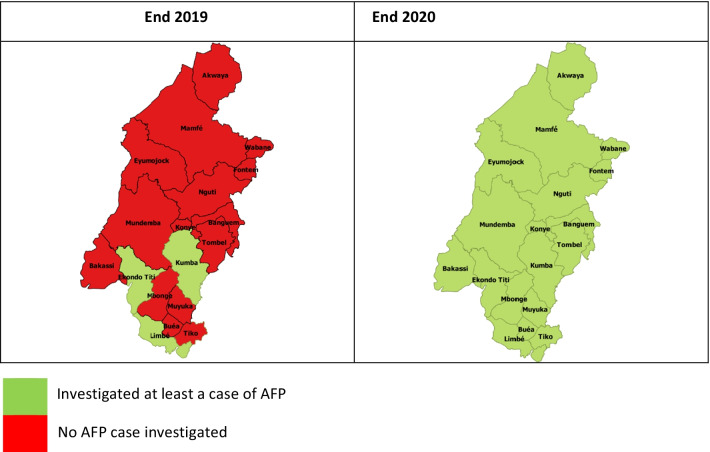
Trend in the investigation of Acute Flaccid Paralysis (AFP) per health district of the Southwest Regions from 2019–2020. A schematic representation of the evolution of AFP investigation in the southwest region of Cameroon between 2019 and 2020. For the corresponding periods, the areas in green are health districts that investigated at least a case of AFP during the given year. Meanwhile, the areas in red are health districts that did not investigate even a case of AFP for the corresponding period

**Fig. 4:**
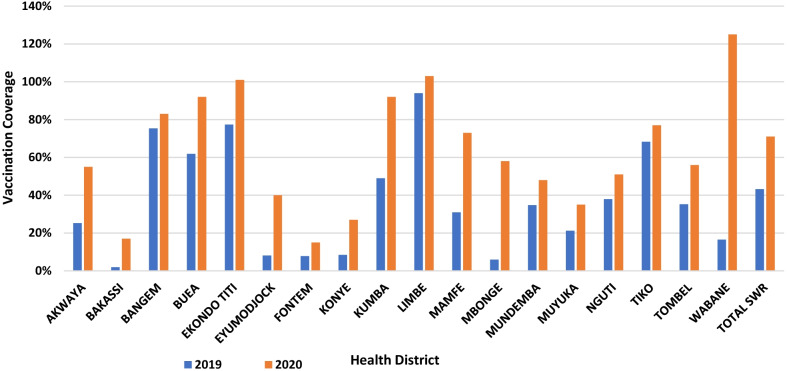
DPT-3 coverage per district 2019 to 2020. The bar chart presents the DPT-3 coverage for the respective health districts of the SW. Blue represents the coverage in 2019 while brown presents the coverage in 2020

The security situation observed in the region in 2020 was similar to what was described for 2019 [19]. It was observed that the number of children vaccinated per month peaked in the months corresponding to the periods when PIRI was carried out (Fig. 2). Every health district experienced an improvement in its performance in 2020 compared to the previous year (Figs. 3 and 4), with the region experiencing a rise in its Penta-3 coverage by 28% points, going from 43% in 2019 to 71.1% in 2020 (Fig. 4). A similar trend was observed for other vaccines given simultaneously (OPV-3 and IPV). Also, the vaccination coverage of MR-1 experienced a 27% points gain, moving from 43.2% in 2019 to 70.5% in 2020.

Furthermore, the BCG experienced a 25%-point improvement, with coverage moving from 48.4% in 2019 to 73.2% in 2020. The annual regional coverage for vaccines newly introduced into the EPI in 2020 stood at 48% for MR-2 and 14% for the first dose of the HPV vaccine. Globally, there was a marked improvement in the vaccination coverage for all the antigens in all the districts compared to the previous 4 years, as initially present in our previous study [19]. Many districts experienced a jump in coverage from 2019 to 2020 (Table 1).

**Table 1:** Trend in immunization equity in the region over 5 years

	South west region				
	2016	2017	2018	2019	2020
*BCG*					
District with VC < 20%, n (%)	0 (0)	0 (0)	2 (11)	1 (5)	1 (5)
District with 20% < = VC < 40%, n (%)	2 (68)	2 (11)	5 (28)	13 (68)	2 (11)
District with 40% < = VC < 60%, n (%)	7 (16)	1(6)	6 (33)	3 (16)	5 (28)
District with 60% < = VC < 80%, n (%)	9 (11)	5 (28)	1 (6)	2 (11)	3 (16)
Districts with VC > = 80%, n (%)	1 (5)	12(67)	4 (22)	0 (0)	7 (39)
*DPT-HepB-Hib*-3					
District with VC < 20%, n (%)	0 (0)	0 (0)	2 (11)	3 (16)	2 (11)
District with 20% < = VC < 40%, n (%)	0 (0)	1 (6)	5 (28)	9 (47)	2 (11)
District with 40% < = VC < 60%, n (%)	3 (16)	1 (6)	6 (33)	3 (16)	4 (21)
District with 60% < = VC < 80%, n (%)	13 (68)	2 (11)	1 (6)	4 (21)	2 (11)
Districts with VC > = 80%, n (%)	3 (16)	14(78)	4 (22)	0 (0)	6 (33)
*MR1*					
District with VC < 20%, n (%)	0 (0)	0 (0)	2 (11)	2 (11)	1 (6)
District with 20% < = VC < 40%, n (%)	0 (0)	2 (11)	6 (33)	10 (53)	4 (22)
District with 40% < = VC < 60%, n (%)	5 (26)	0 (0)	4 (22)	4 (21)	4 (21)
District with 60% < = VC < 80%, n (%)	11 (58)	3 (17)	2 (11)	3 (16)	3 (17)
Districts with VC > = 80%, n (%)	3 (16)	13 (72)	4 (22)	0 (0)	6 (33)

*BCG* bacilli, calmette guerin, *DPT-HepB-Hib* diphtheria, pertussis, tetanus Hepatitis B and hemophilus influenza B, *MR* measles and rubella, *VC* vaccination coverage

Presents the evolution in the vaccination coverage in all the health districts of the southwest per year from 2016 to 2020 for DPT-3, BCG, and MR. The absolute numbers (n) indicate the number of districts whose coverage fall within the respective coverage ranges for each of the vaccines. In brackets are the percentages obtained from the denominator of 18 health districts in the SW in 2020

There was equally an improvement in surveillance indicators in 2020 compared to 2019. In total, 72% of health districts investigated a case of yellow fever in 2020 against 56% in 2019. Also, 66% of these districts investigated a case of measles in 2020 compared to 50% in 2019. It was realized that all 18 health districts investigated at least a case of AFP in 2020 against just 03 in 2019 (Fig. 3).

None of the health workers was reported to have been hurt due to the insecurity. Also, none were reported to have presented with symptoms of COVID-19 during and three weeks following each round of PIRI.

## Discussion

The consequences of the ongoing civil strife in the southwest region of Cameroon have been enormous over the previous 03 years, with a drop in Penta-3 coverage between 2016 (85.5%) and 2019 (45.3%) by 42 percentage points [19]. In addition to the civil strife, the challenges posed by the COVID-19 outbreak that hit the country on March 6, 2020, greatly affected health service delivery in general and immunization services in particular [12–14, 25]. Notwithstanding knowing the benefits of adapting the strategy to the existing challenges [29], the health actors in this region embarked on intensifying routine immunization activities in a context of insecurity and COVID-19.

This study aimed to assess the PIRI’s impact in the SW of Cameroon in 2020 within the context of insecurity and COVID-19 on vaccination service delivery. Interest was in its effect on the coverage of key antigens and some major VPDs surveillance indicators.

There were 54,242 persons vaccinated in the SW following PIRI, which favored an improvement in the vaccination coverage of all antigens. Unlike in 2019, when the region had just 03 districts that investigated a case of acute flaccid paralysis (AFP), in 2020, all 18 health districts examined at least a case of AFP (Fig. 3), with more districts investigating cases of other vaccines preventable disease.

Despite the significant improvement made by the EPI since its creation, it was evident that innovative interventions were relevant in administering routine EPI interventions in this region of Cameroon hit by sociopolitical crisis for over 5 years and COVID-19 in 2020. A significant observation was the killing of health workers, lotting of health infrastructures, and mass population displacement [19]. So, the EPI staff of this region and its 18 health district managers worked closely with the community to identify physically healthy residents and accepted in their respective communities to help ensure the continuity of health services like vaccination and disease surveillance. This community-led approach helped adopt an intervention that downplayed the impact of local challenges like insecurity [29] and improved vaccine uptake and disease surveillance performances. PIRI, in this case, allowed the health workers to reach a population that had been partially served for more than 4 years following insecurity. The primary intervention was through mobile teams getting the people in their homes and internally displaced sites rendering healthcare services at the site and time of convenience; this improved vaccine uptake [30]. This approach ties with the WHO recommendations [30]. With the prevailing insecurity in the region for the previous 4 years, the healthcare system was disrupted. This disruption resulted from mass population movement, the killing of health staff, looting of health facilities, and interference with transport services [19]. The deterioration in health indicators in this conflict region is like observations in other countries across the globe that faced armed conflict [15, 31].

COVID-19 was an added burden to this already weakened health system. With the emergence of this disease, there was a global disruption of health service delivery. There were curfews, restrictions of movements, and mass interventions, including access to healthcare and outreach vaccination services globally and in Cameroon [20, 32]. This pandemic further caused millions of children to miss their vaccine doses and favored outbreaks worldwide, particularly in the African Region [9, 32]. Despite these restrictions due to COVID-19 in Cameroon and the WHO African Region at large in 2020 [33], PIRI and other routine immunization activities enabled the southwest region of Cameroon to vaccinate 37,673 infants with the third dose of Oral Polio Vaccine (OPV-3) while respecting the majors put in place to limit the spread of COVID-19. This intervention helped the SW OPV-3 coverage to improve by 28 percentage points gained that year. So, bringing the community to action helped the region attain its objective in the fight against poliomyelitis amid the crisis [34].

Conflicts have a negative consequence on immunization services. Armed violence interrupted service delivery, and commodity flow channels disrupted cold chain maintenance and favored population movement [15, 19, 31]. The challenges routinely posed by conflict have been preexisting in this region since 2016 [19]. This innovative strategy adapted to the local realities was helpful in this region that faced a double burden of insecurity and COVID-19. The intervention helped the actors to make significant progress in all major EPI indicators as recommended by the World Health Organization (WHO) [30]. Also, newly introduced vaccines like MR-2 and HPV experienced a rapid uptake. For instance, MR-2 vaccination coverage reached 48% in just nine months following its introduction in March 2020.

Moreover, the MR-2 performance got close to the target set by the WHO 2020 [35]. Also, HPV annual coverage reached 11% two months after its introduction in October 2020. This improvement was a significant milestone in introducing the HPV vaccine [35], protecting several adolescent girls from human papillomaviruses in the region and thus cervical cancer. The health workers used to implement PIRI in the communities were locally recruited and resided in the respective communities within insecurity. This approach of recruiting community health workers helped improve access and penetration into the community and ensured the delivery of these life-saving healthcare services [30]. These results were possible thanks to the interventions of PIRI, which was used to improve coverage of traditional vaccines and helped to introduce new vaccines, even in this crisis setting [35–37].

Also, respecting the guideline set to ensure continuity of immunization and other healthcare services helped the actors effectively carry out vaccination and active disease surveillance amid the COVID-19 pandemic. Though COVID-19 control measures had negative consequences on vaccination globally and Cameroon [3, 13, 33, 39], PIRI helped the SW downplay the impact on EPI indicators. Notwithstanding, the first case of COVID-19 was reported in Cameroon in March 2020, and this region investigated its first case about a month later [38]. Though fewer disease cases were reported in SW, the prevalence of the COVID-19 continued to increase every week [13, 20, 21].

Even though none of the health workers involved in PIRI presented with COVID-19 symptoms during and three weeks following PIRI, the risk of COVID-19 spread remained. Moreover, in early 2020, screening for COVID-19 was not common, especially in the rural communities of Cameroon [20]. Also, at the onset, polymerized chain reaction (PCR) was the primary test done in three diagnostic sites located in the center and littoral regions of the country [39].

The community health workers (CHW) used during PIRI were trained on disease surveillance. This synergy between immunization and surveillance activities helped favor investigating at least a case of AFP in all 18 health districts in 2020 compared to 2019, when just three health districts investigated a case. This AFP surveillance improvement partly contributed to attaining the goals of the Global Polio Eradication Initiative, even in this area of insecurity in 2020 [24]. Notwithstanding, though an improvement was observed in the proportion of health districts that investigated at least a case of the other VPDs, this improvement was more remarkable with AFP surveillance. This high detection of AFP cases can be linked to the attention paid to polio surveillance as the country and the African continent aimed to be certified free of wild poliovirus in 2020 [33, 40].

With the existing EPI platform in this region, the targeted population benefits daily from vaccination and surveillance services at no additional cost to the health system. Staff in the vaccinating facilities depend on their routine wages from their employer for the services rendered to the population. The implementation of PIRI increased the intervention cost by 4.50 USD per person vaccinated. This was an added cost, but it was worth it. During this period of PIRI in the SW, several parts of the world experienced a drop in vaccination coverage and some outbreaks from VPDs [3, 9, 32]. This region rather enjoyed an improvement in its vaccination coverage. Though more suspected cases of the major disease under surveillance were investigated, an outbreak of any of these diseases was not captured in 2020.

Before 2016, this region of Cameroon enjoyed high EPI performances, with DPT-3 coverage standing above 80% [19]. With the onset of the conflict, these indicators dropped with the worst values recorded in 2019 [19]. PIRI did not just maintain but improved vaccination and surveillance performance even with the additional challenge of COVID-19 [3, 7, 8]. Though Cameroon and other parts of the world experienced hesitance and limited access to routine immunization, PIRI helped the SW to experience improvement [8, 9, 13, 32].

This is the first report of PIRI’s contribution to immunization and disease surveillance amid insecurity and the COVID-19 in the SW of Cameroon. Despite this merit, this piece of work had some limitations. Firstly, the primary data was collected in a context of insecurity, which could have introduced some bias into the data. Also, the denominator used to calculate coverages was based on estimates from the central level, which may not necessarily have reflected the actual situation on the ground given the population movement reported in this area since the onset of the crisis [25]. Finally, due to the subjective registered COVID-19 status based on the COVID-19 related signs and symptoms reported by the actors involved in the activity, some cases of COVID-19 may have occurred within the communities linked to PIRI, but this could have been missed. Despite these limitations, we firmly believe that this study sets the scene for a preliminary understanding of the impact of PIRI in this setting in improving vaccination and surveillance performances in the context of insecurity and the COVID-19 pandemic. We remain hopeful that this study will stimulate more research to elucidate the benefit of PIRI in the COVID-19 pandemic and clearly state the risk associated with carrying out PIRI in the current COVID-19 era and propose adequate mitigating strategies.

## Conclusion

The findings of this study suggest that PIRI improved the performance of routine vaccination coverage and disease surveillance of VPDs in the SW of Cameroon in the context of insecurity and COVID-19. PIRI also helped rapidly stimulate the uptake of newly introduced vaccines like the MR-2 and HPV vaccines. These findings suggest that PIRI is a valuable strategy for rapidly improving vaccine uptake and surveillance performances. It equally means that if health actors respect the recommended policies, PIRI can help rollout new vaccines rapidly, particularly in an emergency context such as those posed by the COVID-19 pandemic and the armed crisis in the SW of Cameroon.

## Data Availability

The datasets used during this study are available from the corresponding author on reasonable request. Part of this data can also be directly obtained from the Dhis 2 database of this region.

## Abbreviations

BCG: Bacilli, calmette guerin
Covid: Corona virus diseases
CHW: Community health worker
DVDMT: District vaccine and data management tool
DPT-HepB-Hib: Diphtheria, pertussis, tetanus hepatitis B and hemophilus influenza B
HPV: Human papilloma virus
IPV: Inactivated polio vaccine
MR: Measles and rubella
OPV: Oral polio vaccine
PCV: Pneumococcal conjugate vaccine
PCR: Polymerized chain reaction
PIRI: Periodic intensification of routine immunization
SW: South west region
SARS COV 2: Severe acute respiratory syndrome corona virus two
UNICEF: United nations children fund
WHO: World health organization
VPDs: Vaccine-preventable diseases
Td: Tetanus-diphtheria
